# Spatial RNA velocity reveals cellular state transitions and prognostic markers in the melanoma microenvironment

**DOI:** 10.3389/fonc.2026.1845013

**Published:** 2026-06-22

**Authors:** Xiangjian Fang, Juntao Cheng, Zhiyi Wei, Biao Wang

**Affiliations:** 1 Department of Plastic and Aesthetic Surgery, the First Affiliated Hospital of Fujian Medical University, Fuzhou, Fujian, China; 2 Department of Burn and Wound Repair, Quanzhou First Hospital Affiliated to Fujian Medical University, Quanzhou, Fujian, China

**Keywords:** biomarkers, melanoma, RNA velocity, spatial transcriptomics, tumor-infiltrating T cells

## Abstract

The inability to infer transcriptional dynamics from high-resolution spatial transcriptomics represents a major computational challenge, as these datasets lack the spliced/unspliced mRNA counts required for conventional RNA velocity. To address this, we developed a novel computational framework that repurposes subcellular transcript localization—using nuclear and cytoplasmic RNAs as proxies for unspliced and spliced mRNA, respectively—for spatial RNA velocity analysis. By integrating this approach with scVelo’s dynamical model, we inferred directional state transitions directly from melanoma spatial transcriptomes. Our framework successfully reconstructed progression trajectories of melanoma cells and differentiation paths of infiltrating T cells, identifying cluster-specific dynamic genes. These genes were significantly associated with patient prognosis and formed protein-protein interaction networks enriched for immune-related pathways. This study provides a generalizable computational strategy to decode spatiotemporal dynamics from static spatial transcriptomic data, bridging a critical gap between spatial biology and transcriptional dynamics.

## 1 Introduction

Spatial transcriptomics has rapidly transformed our ability to characterize tissue architecture by mapping gene expression with preserved spatial context, revealing complex cellular organization and microenvironmental niches across many tumor types (1–3). Early sequencing- and imaging-based spatial platforms demonstrated that tumors are composed of intermixed malignant, immune, stromal and vascular compartments with spatially restricted programs and microanatomical neighborhoods that correlate with function and pathology (1, 4, 5).

Melanoma is a prototypical example in which cellular heterogeneity and immune infiltration strongly influence progression and therapeutic response (6–8). Single-cell RNA-seq studies uncovered transcriptionally distinct malignant cell states, stromal and myeloid programs, and diverse lymphocyte populations that together shape tumor behavior and treatment outcomes (9, 10). Clinical studies have further documented genetic and microenvironmental mechanisms of resistance to immune checkpoint blockade - examples include loss-of-function alterations in antigen-presentation machinery (e.g., B2M) and adaptive changes in tumor-immune signaling -underscoring the need to link cellular state, spatial context, and clinical phenotype.

RNA velocity analysis provides a framework to infer short-term transcriptional dynamics and putative future states from single-cell RNA-seq data by exploiting the balance between unspliced (nascent) and spliced (mature) transcripts, which unveils the dynamics of transient cells during pathogenesis (11, 12). The original velocity concept and software established the idea of per-cell “velocity vectors” that predict transcriptional change, and later methodological advances (notably the dynamical model implemented in scVelo) generalized velocity inference to transient and complex trajectories (11, 13). These tools have since been incorporated into many single-cell workflows to reconstruct differentiation trajectories and dynamic responses.

Despite these advances, a key methodological gap persists: standard spatial transcriptomic assays (both sequencing-based and many imaging approaches) do not routinely provide the canonical unspliced/spliced readouts used for RNA velocity, preventing direct application of existing velocity frameworks to intact tissue maps. At the same time, newer high-plex, subcellular spatial imaging platforms (e.g., CosMx SMI and related spatial molecular imaging systems (14, 15)) produce single-cell and subcellular localization of transcripts and proteins, suggesting a route to reconstruct kinetic proxies from spatially resolved data. These technological and analytical developments motivate strategies that exploit subcellular transcript localization (nuclear vs cytoplasmic) to approximate unspliced and spliced pools and thereby extend velocity concepts into the spatial domain. However, such proxy-based approaches rely on simplifying assumptions regarding RNA processing and localization, and therefore require careful interpretation when inferring transcriptional dynamics from spatial data.

Here, we develop and apply a spatial RNA velocity framework that leverages nuclear and cytoplasmic transcript signals as practical proxies for unspliced and spliced mRNA in high-resolution spatial datasets. After segmentation and compartmentalized transcript quantification, we integrate these proxies with established velocity pipelines (scVelo and downstream trajectory tools) to infer latent time and directed transitions within tissue sections. We then apply this approach to deconvolve melanoma cell states and infiltrating T cell trajectories separately, identify cluster-specific dynamic marker genes, and interrogate the functional and clinical relevance of those markers using pathway enrichment and prognostic analyses. Methodologically, our workflow builds on prior single-cell/spatial integration strategies (e.g., Seurat anchoring, ST and scRNA integration) and on downstream tools for velocity-informed trajectory extraction.

By combining subcellular spatial readouts with dynamical modeling, this study provides a spatially resolved view of transcriptional kinetics in melanoma. Our results reveal distinct spatially patterned progressions within malignant cells, parallel differentiation trajectories among tumor-infiltrating T cells, and dynamic marker genes (including immune-related factors) that associate with patient prognosis and form coherent interaction networks. This spatial-dynamic perspective complements and extends existing single-cell and spatial atlases, offering a new analytical avenue to connect tissue architecture, cellular dynamics, and clinical outcome.

## 2 Methods

### 2.1 Data sources and overview

High-plex spatial transcriptomic data (16) (CosMx SMI 1k) from melanoma tissues were used for all analyses (https://datadryad.org/dataset/doi:10.5061/dryad.ksn02v7b1). Raw per-molecule transcript tables and associated cell segmentation/metadata were ingested as CSV files and processed to produce cell-level expression matrices for subcellular compartments (nucleus, cytoplasm, membrane) as described below. Downstream analyses were performed in Python using Scanpy, scVelo, loompy, pandas and matplotlib (code available from the corresponding authors on request).

### 2.2 Preprocessing and quality control

Raw transcript tables containing at least the fields cell, target (gene), and CellComp (subcellular compartment) were read in chunked mode to conserve memory. For each compartment (Cytoplasm, Nuclear, Membrane) we aggregated counts per (cell, gene) pair to produce compartment-specific count matrices. Matrices were exported as CSV files and subsequently read into AnnData objects for analysis.

Initial normalization was applied for exploratory analysis, while velocity inference strictly followed the scVelo preprocessing pipeline. The 100 genes used for velocity inference were selected as a subset of highly variable genes (HVGs), ensuring consistency with upstream preprocessing.

For compartment- or sample-level analyses, count matrices were normalized using total-count normalization (sc.pp.normalize_total(target_sum=1e4)), log-transformed (sc.pp.log1p) and filtered to remove lowly expressed features (e.g., sc.pp.filter_genes(min_cells=2), sc.pp.filter_cells(min_genes=2) as applied). Highly variable genes (HVGs) were selected with sc.pp.highly_variable_genes; for whole-sample analyses we used n_top_genes=2000, while for some subpopulation workflows n_top_genes=1000 (we note these parameter choices in the text and code; they may be tuned depending on dataset size). Principal component analysis (PCA) was computed with sc.tl.pca(n_comps=30) and neighborhood graphs were constructed with sc.pp.neighbors (typical settings: n_pcs=30, n_neighbors=15–30 depending on analysis). UMAP coordinates were computed using sc.tl.umap.

We quantified subcellular transcript distributions across nuclear, cytoplasmic, and membrane compartments. Across all detected transcripts, nuclear-localized RNAs accounted for 62.13%, cytoplasmic transcripts for 23.79%, and membrane-associated transcripts for 14.08%. Given the heterogeneous biological interpretation and potential extracellular or boundary-associated noise of membrane-localized transcripts in imaging-based spatial transcriptomics, we excluded membrane counts from downstream RNA velocity modeling and focused on nuclear and cytoplasmic compartments as the primary proxy for transcriptional dynamics.

### 2.3 Cell clustering and annotation

Leiden clustering was performed with sc.tl.leiden(…, resolution=0.5) to partition cells into transcriptionally coherent clusters. Marker genes for each cluster were identified with Wilcoxon rank-sum testing via sc.tl.rank_genes_groups(method=‘wilcoxon’) and exported using sc.get.rank_genes_groups_df.

To produce biologically interpretable cell-type annotations we defined a curated list of canonical marker genes for major cell types (T cell, B cell, melanoma, normal endothelial/mesenchymal). For each cell we computed normalized scores for each marker set and then calculated the mean expression of the marker set within each Leiden cluster. Cells with a mean marker score <0.1 across marker gene sets were labeled as “unknown/mix” to avoid ambiguous assignments when marker signals were weak or overlapping. This threshold was empirically chosen to balance annotation specificity and coverage, and moderate variations in the cutoff produced similar cell-type distributions. The mapping was written into adata.obs[‘cell_type’]. Dotplots and UMAPs showing marker expression and cell-type scores were generated with Scanpy plotting functions and saved as high-resolution PNGs.

### 2.4 Generation of spliced/unspliced proxy matrices (nucleus-cytoplasm strategy

Velocity models were independently fitted after lineage-specific subsetting, ensuring that dynamical parameters were not shared across distinct cellular compartments. To enable RNA-velocity-style analysis from imaging-based spatial data, we treated transcripts localized to the cytoplasm as proxies for “spliced-like” (late) RNA and transcripts localized to the nucleus as proxies for “unspliced-like” (early) RNA. This strategy leverages the general biological principle that nascent transcripts are initially retained in the nucleus before export to the cytoplasm. Nevertheless, we acknowledge that RNA localization is influenced by multiple factors, including transcript processing kinetics, nuclear retention, and RNA stability. Therefore, the nucleus-cytoplasm partitioning should be interpreted as an approximate proxy rather than a direct measurement of spliced and unspliced transcripts.

From the compartment counts, we selected the intersection of cells and genes present in both nuclear and cytoplasmic matrices and reindexed matrices to these common cells/genes. Using loompy, we created a Loom file that included spliced and unspliced layers (cytoplasm and nucleus counts, respectively) and generated an AnnData object with these layers for downstream scVelo analysis. This proxy is intended to capture aggregate transcriptional trends across genes rather than exact gene-level kinetics.

### 2.5 Downstream clustering and marker discovery for subpopulations

Following creation of the AnnData object with compartment layers, data were further processed for subpopulation-level analyses. Standard Scanpy preprocessing (filtering, normalization, log1p, HVG selection) was applied. Dimensionality reduction (PCA with 30 components) and neighborhood graph construction (typical n_neighbors = 30, n_pcs = 30) preceded Leiden clustering (resolution = 0.5) and UMAP visualization. Cluster marker genes were identified using sc.tl.rank_genes_groups and visualized with sc.pl.rank_genes_groups and dotplots.

These parameters can be flexibly adjusted depending on dataset characteristics and analysis goals, and may influence local velocity structure.

### 2.6 Spatial RNA-velocity inference (scVelo dynamical model

Velocity preprocessing followed the scVelo recommended pipeline with settings adapted to the proxy matrices:

1. Gene/cell filtering and normalization: scv.pp.filter_and_normalize(adata, min_shared_counts=1, n_top_genes=100) (parameter values adjusted as needed).
2. Moment calculation: scv.pp.moments(adata, n_pcs=30,n_neighbors=30).
3. Dynamical model fitting: scv.tl.recover_dynamics(adata) followed by scv.tl.velocity(adata, mode=‘dynamical’).
4. Velocity graph computation: scv.tl.velocity_graph(adata).
5. Latent time estimation: scv.tl.latent_time(adata) (computed after recover_dynamics).
6. Velocity inference was performed separately for melanoma cells and T cells to avoid mixing transcriptional dynamics across distinct cellular lineages. Velocity vectors and latent-time values were saved in the AnnData object and exported to disk (adata.write) for record-keeping. Velocity fields were visualized either on embedding spaces (UMAP) via scv.pl.velocity_embedding_stream(…, basis=‘umap’) or directly on tissues via scv.pl.velocity_embedding_stream(…, basis=‘spatial’) after assigning spatial coordinates into adata.obsm[‘spatial’]. For spatial plotting we aligned cell identifiers between adata.obs.index and the external metadata file (containing spatial_x and spatial_y) and added those columns to adata.obs. Stream/arrow plotting parameters (e.g., arrow_size, density, size) were set to yield publication-quality figures and saved as high-resolution PNG files.

To assess robustness to segmentation uncertainty, small perturbations were introduced to compartment-specific transcript counts, followed by re-estimation of velocity. The inferred latent time remained highly consistent with the original results (Pearson correlation R = 0.954).

### 2.7 Differential expression, pathway and enrichment analyses

Cluster-specific marker genes selected by sc.tl.rank_genes_groups were used for downstream pathway analysis. Enrichment analyses were performed using Metascape with default settings to identify enriched GO Biological Processes and KEGG pathways for cluster marker gene lists. Results reported in the manuscript (e.g., extracellular matrix degradation, leukocyte activation, Wnt signaling, cytokine–receptor interactions, oxidative stress response) correspond to significantly enriched terms (adjusted p-value threshold per Metascape defaults).

### 2.8 Prognostic analysis and network construction

To assess clinical relevance, candidate dynamic marker genes (union of melanoma- and T cell-derived markers) were evaluated for expression associations across TCGA cohorts (>20 cancer types) using Gepia2 (http://gepia2.cancer-pku.cn/#survival). For Kaplan-Meier analyses, patients were dichotomized into high and low expression groups (median split) and overall survival differences were tested using the log-rank test. Representative examples (e.g., *B2M*, *CD74*) are shown in the manuscript. Protein–protein interaction networks were constructed using STRING (string-db.org) with standard confidence thresholds and visualized as interaction maps. NetworkAnalyst was used to generate additional network visualizations and identify central hubs and subnetworks. KEGG and GO-BP enrichment for the marker gene set were reported alongside PPI analyses.

### 2.9 Cell culture and transfection

Human melanoma cell line A375 (SCSP-533) and murine melanoma cell line B16 (TCM 2) were purchased from and authenticated by the National Collection of Authenticated Cell Culture (https://www.cellbank.org.cn/). Cells were cultured in Dulbecco’s Modified Eagle Medium (DMEM, Gibco) supplemented with 10% fetal bovine serum (FBS, Gibco) and 1% penicillin/streptomycin (Gibco) at 37 °C in a humidified incubator with 5% CO2.

For CD74 knockdown, cells were transfected with either CD74-targeting siRNA (siCD74) or a non-targeting control siRNA (siNC) using Lipofectamine™ RNAiMAX according to the manufacturer’s instructions. The transfection efficiency was verified by quantitative real-time PCR (qPCR) and immunoblotting 48–72 hours post-transfection. For the *in vivo* mouse model, CD74 was stably knocked down using a lentivirus expressing an shRNA designed to generate the same siRNA duplex as siCD74-1. A non-targeting shRNA control was used in parallel. All cell lines were routinely tested and confirmed to be free of mycoplasma contamination prior to use.

siRNA siCD74-1: CCAGGACCAUGUGAUGCAUTT
siRNA siCD74-2: UUCUCCGAACGUGUCACGUTT
siRNA siNC: UUCUCCGAACGUGUCACGUTT
PCR primers:
CD74-F: GACGAGAACGGCAACTATCTG
CD74-R: GTTGGGGAAGACACACCAG
ACTB-F: CTCTTCCAGCCTTCCTTCCT
ACTB-R: AGCACTGTGTTGGCGTACAG

### 2.10 RNA extraction, quantitative real-time PCR, and immunoblotting

Total RNA was extracted from cells using TRIzol reagent (Invitrogen). cDNA was synthesized using a PrimeScript RT reagent kit (Takara). qPCR was performed on a QuantStudio system (Applied Biosystems) using SYBR Green PCR Master Mix (Takara). β-Actin was used as an internal control for mRNA expression.

For immunoblotting, cells were lysed in RIPA buffer supplemented with protease inhibitors. Proteins were separated by SDS-PAGE, transferred to NC membranes, and incubated with primary antibodies against CD74 and GAPDH overnight at 4 °C, followed by incubation with HRP-conjugated secondary antibodies. Protein bands were visualized using an enhanced chemiluminescence (ECL) detection system.

For reverse transcription, 1 ug of total RNA was converted to cDNA using the RT reagent kit (Takara) under the following conditions: 37 °C for 15 min, followed by 85 °C for 5s to inactivate the reverse transcriptase.

qPCR was performed using SYBR Green PCR Master Mix (Takara) on a QuantStudio Real-Time PCR System (Applied Biosystems). The thermocycling conditions were as follows: initial denaturation at 95 °C for 10 minutes, followed by 40 cycles of: 95 °C for 10s, 60 °C for 20s and 72 °C for 20s.

### 2.11 Cell proliferation and viability assays

Cell Counting Kit-8 (CCK-8) was used to assess cell viability. At 24 hours post-transfection, cells were seeded into 96-well plates. At the indicated time points, CCK-8 reagent was added to each well and incubated for 2 hours. The absorbance at 450 nm was measured using a microplate reader.

### 2.12 EdU staining

DNA replication activity was measured using a EdU staining kit according to the manufacturer’s protocol. Briefly, cells were seeded into 24-well plates and incubated with 50 μM EdU for 0.5 hour at 37 °C. After fixation and permeabilization, cells were stained with Apollo567 reaction cocktail and counterstained with DAPI to visualize all nuclei. The count of EdU-positive cells was calculated from images captured by a fluorescence microscope.

### 2.13 Transwell invasion assay

Cell invasion was evaluated using 24-well Transwell chambers (Corning) coated with Matrigel (BD Biosciences). At 48 hours post-transfection, 5×10^4^ cells in serum-free medium were seeded into the upper chamber. The lower chamber was filled with medium containing 20% FBS as a chemoattractant. After 24–48 hours of incubation, non-invading cells on the upper surface of the membrane were removed with a cotton swab. Invaded cells on the lower surface were fixed with 4% paraformaldehyde, stained with 0.1% crystal violet, and photographed and counted under a microscope in five random fields.

### 2.14 Visualization and figure generation

Figures were generated using Scanpy, scVelo and matplotlib with figure sizes and DPI adjusted for publication quality. Dotplots, heatmaps, UMAPs, velocity streams and spatial velocity overlays were exported as high-resolution PNG files and organized into figure panels corresponding to manuscript figures.

### 2.15 Data and code availability

All scripts used for data preprocessing, compartmentalized count aggregation, Loom generation, clustering, velocity inference, enrichment, and survival analyses are available from the corresponding authors on reasonable request. Processed AnnData and Loom files supporting the results are archived on institutional servers and will be made available upon publication subject to data sharing policies. We used a high-plex spatial transcriptomic data (16) (CosMx SMI 1k) from melanoma tissues in the study and the raw data was previously deposited at DRYAD (https://datadryad.org/dataset/doi:10.5061/dryad.ksn02v7b1).

### 2.16 Statistical analysis

All data are presented as the mean ± SEM from at least three independent experiments. Statistical analyses were performed using GraphPad Prism software (Version 9.0). Differences between two groups were analyzed using the unpaired two-tailed Student’s t-test. A p-value of less than 0.05 was considered statistically significant (*p < 0.05, **p < 0.01, ***p < 0.001).

Differential gene expression analysis in Scanpy was performed using the Wilcoxon rank-sum test. Survival differences were assessed using the log-rank test. Multiple testing correction was performed using the Benjamini-Hochberg method where appropriate. All tests were two-sided, and p < 0.05 2was considered statistically significant.

### 2.17 Animal model

Ethics. Mice were housed under SPF conditions (22 ± 2 °C, 12 h light/dark cycle) with free access to food and water. All procedures adhere to the ARRIVE guidelines and were approved by the Institutional Animal Care and Use Committee (IACUC) of Animal Ethics Committee of the Medical College, Huaqiao University. Ethic number is A2025080.

Quanzhou First Hospital Affiliated to Fujian Medical University does not currently have a qualified SPF animal facility for *in vivo* mouse experiments. Therefore, all animal experiments were conducted at the Animal Facility of the Medical College, Huaqiao University, which provides a certified SPF platform. Our institution was fully informed of and approved the use of this external facility for the animal experiments.

Animal study design. Fifteen male C57BL/6 mice (6–8 weeks old) were randomly assigned into three groups (n = 5 per group): control (parental B16 cells), shCtrl, and shCD74. Each mouse was subcutaneously injected in the right flank with 1 × 1^6^ B16 melanoma cells in 100μL PBS. Tumor growth was monitored every 2 days using digital calipers, and animals were observed daily for general health status, including activity level, mobility, grooming condition, posture, and food and water intake. Body weight was recorded regularly.

Humane endpoints were predefined before the start of the experiment. Mice were humanely euthanized if tumor volume exceeded 1000 mm^3^, if ulceration or necrosis occurred at the tumor site, if body weight loss exceeded 20%, or if animals showed signs of distress, including lethargy, reduced mobility, or inability to access food and water.

Mice were euthanized by CO_2_ inhalation followed by cervical dislocation to ensure death. No additional agents were administered.

For the survival analysis, we injected the B16 cells stably expressing shCtrl and shCD74, and observed the survival of mice for 20 days. And mice with xenografts exceeding 1000 mm^3^ were sacrificed.

Lentiviral knockdown of CD74. Murine B16 melanoma cells were cultured in DMEM with 10% FBS. Lentiviral vectors carrying shRNA targeting CD74 (sequence identical to previously reported constructs) or a non-targeting control were packaged in HEK293T cells and used to infect B16 cells. Stable transductants were selected with puromycin and verified for knockdown efficiency by qRT-PCR and Western blotting.

Analysis. Statistical significance was determined by Student’s t-test using GraphPad Prism 9.0; p < 0.05 was considered significant.

### 2.18 Immunoblotting

Primary antibody targeting CD74 (Cell Signaling Technology, D5N3I, #77274) and beta-Actin (Cell Signaling Technology, 13E5, # 4970) was used in this study. Primary antibody was incubated overnight at 4 degrees and dilution for beta-Actin is 1:1000 and for CD74 is 1:500. An Anti-rabbit IgG, HRP-linked Antibody (Cell Signaling Technology, #7074) was used as secondary antibody and diluted at 1:2000. Secondary antibody was incubated at room temperature for 1 hour.

## 3 Results

### 3.1 Spatial transcriptomics reveals the cellular composition of melanoma tissues

**Figure 1:**
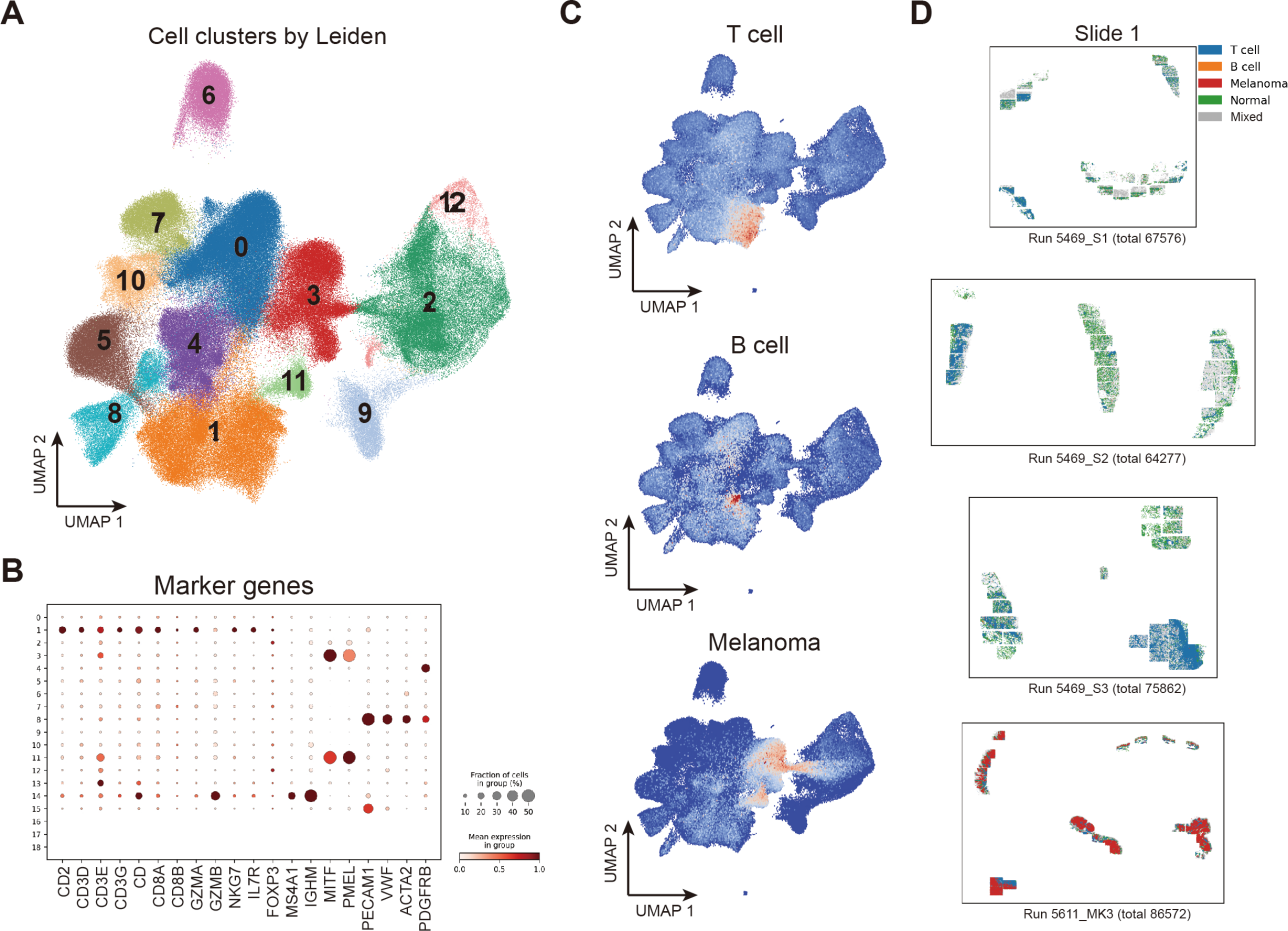
Spatial transcriptomics showing the component of different cell types in melanoma tissues. **(A)** Leiden clustering showing different cell clusters in melanoma tissues from CosMx SMI 1k spatial transcriptomics. **(B)** Dot plot showing the marker genes for distinct leiden clusters. **(C)** Heatmap showing the distribution of T cells, B cells and Melanoma cells in melanoma patients. **(D)** Spatial distribution of T cells, B cells and Melanoma cells in melanoma patients.

To chart the cellular landscape of melanoma, we aggregated CosMx SMI 1k spatial transcriptomic data across samples and performed Leiden clustering on the pooled cells. Clustering partitioned the dataset into 18 transcriptionally distinct clusters (**Figure 1A**), and marker genes for each cluster were identified (**Figure 1B**). Based on published markers and biological priors, we defined gene sets to annotate major cell types and focused our downstream analyses on T cells, B cells, normal (endothelial/vascular) cells and melanoma cells. The principal markers used were: T cells: CD8A, CD2, CD3D, GZMA, GZMB, CD3E, CD3G, NKG7, IL7R, CD4; B cells: MS4A1, IGHM; melanoma: MITF, PMEL; normal (endothelial/vascular): PECAM1, VWF. For each cell we computed normalized scores for these marker sets; cells whose highest marker score was < 0.1 were labeled as mix/unknown, while remaining cells were assigned to the cell type with the maximum score. Using this approach we successfully separated melanoma cells from infiltrating immune and normal cells (**Supplementary Figures 1A, B**). The spatial distributions of T cells, B cells, melanoma cells and normal cells are shown in **Figures 1C, D**, revealing regionally enriched microenvironmental niches and localized immune infiltrates (**Supplementary Figure 2**). These results establish a high-confidence cellular annotation and spatial map that form the basis for subsequent dynamic analyses.

### 3.2 Typical gene expression patterns and establishment of a spatial RNA-velocity model

**Figure 2:**
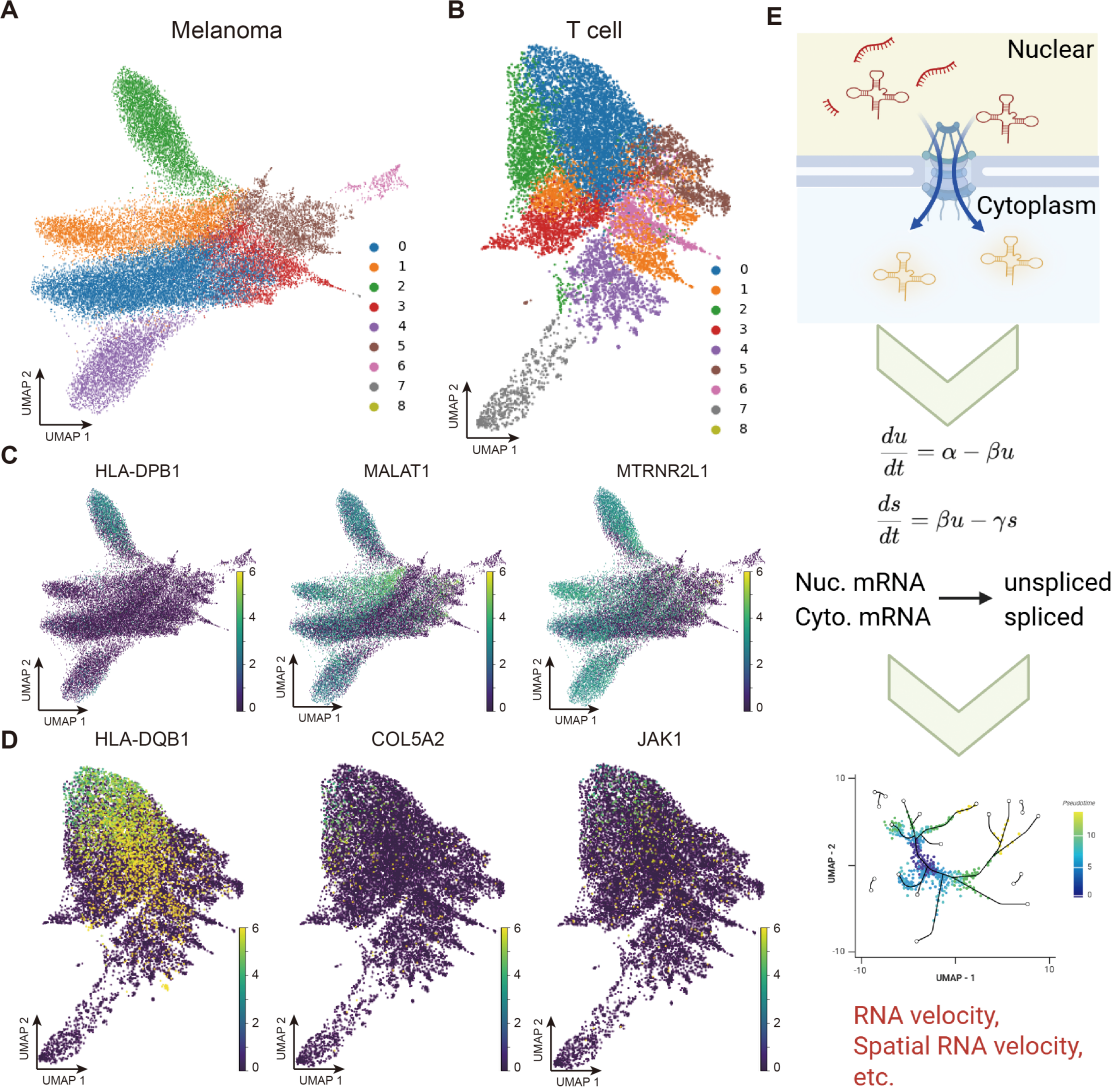
Typical gene expression in melanoma patients and the establishment of spatial RNA velocity. **(A, B)** Leiden clustering showing distinct component in melanoma **(A)** and T cell infiltration **(B)** in melanoma patients. **(C, D)** Heatmap showing the expression of markers for different clusters of melanomas **(C)** and T cells **(D, E)** Schematic figure showing the establishment of spatial RNA velocity in melanoma patients. Briefly, nuclear transcripts were used as proxies for unspliced (nascent) mRNA and cytoplasmic transcripts as proxies for spliced (mature) mRNA in the RNA velocity analysis.

We next subdivided the annotated melanoma and T cell compartments and performed independent Leiden clustering within each compartment to resolve intra-population heterogeneity (**Figures 2A, B**). Differential expression analysis within these sub-clusters identified distinctive marker genes: examples in melanoma included HLA-DPB1, MALAT1, and MTRNR2L1 (**Figure 2C**), whereas T cell clusters were marked by genes such as HLA-DQB1, COL5A2, and JAK1 (**Figure 2D**; **Supplementary Figure 3**). These signatures reflect both intrinsic malignant states and functional/activation programs among infiltrating lymphocytes.

Because conventional spatial transcriptomic assays do not directly provide the canonical unspliced/spliced counts required for RNA-velocity, we developed a spatial RNA-velocity strategy that uses subcellular transcript localization as kinetic proxies (schematic in **Figure 2E**). Following cell segmentation and compartmentalized transcript counting, transcripts localized to the nucleus were treated as proxies for “early” (unspliced-like) mRNA and transcripts localized to the cytoplasm as proxies for “late” (spliced-like) mRNA. By analogy to RNA-velocity’s use of unspliced to spliced dynamics, these nucleus to cytoplasm proxies were used as inputs to the dynamical model implemented in scVelo. After normalization and model fitting, the framework yields per-cell velocity vectors and latent time estimates in the spatial context, enabling inference of likely future transcriptional states across the tissue. Applying this pipeline permits dynamic, spatially resolved predictions of cell-state transitions in high-resolution spatial data; subsequent sections apply the model to reconstruct melanoma progression trajectories and T cell differentiation dynamics.

### 3.3 scVelo analysis reveals distinct markers and trajectories of melanoma progression

**Figure 3:**
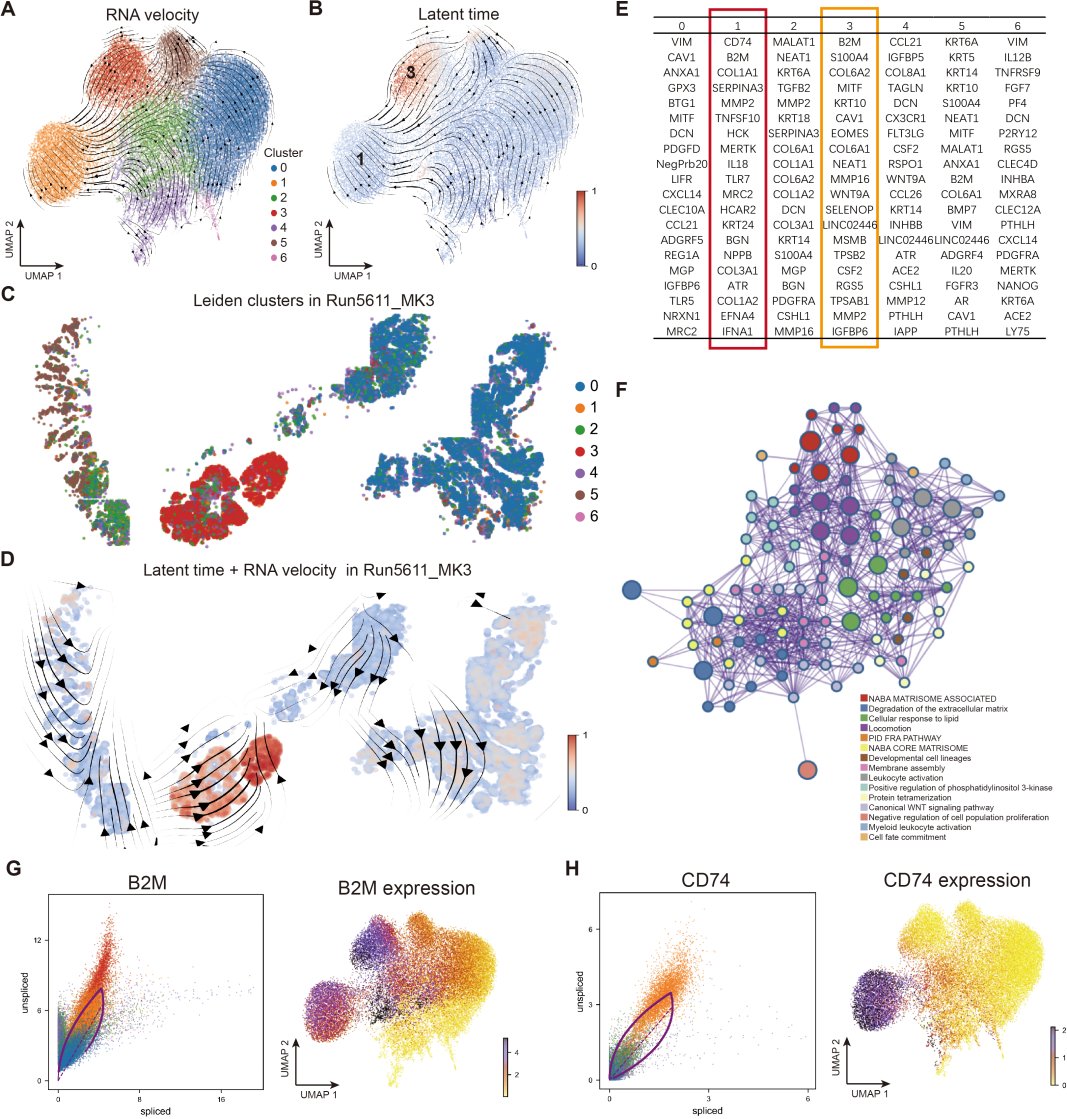
scVelo analysis showing distinct markers for melanoma progression. **(A, B)** Leiden clustering **(A)** and latent time **(B)** in melanoma cells. RNA velocity was calculated with the model established in **Figure 2E** and imposed on Leiden and latent time results. **(C)** Spatial distribution of melanoma cells from distinct Leiden clusters. **(D)** Heatmap showing the spatial distribution of cells from distinct latent time. RNA velocity was calculated from scVelo and imposed on the latent time. **(E)** Tables showing markers for different Leiden clusters. **(F)** Metascape analysis showing the pathway enrichment with marker genes in Leiden cluster 1 and cluster 3. **(G, H)** Dot plot showing the spliced/unspliced levels in melanoma cells **(G)** and heatmap showing expression of marker genes for cluster 1 and cluster 3 **(H)**.

Building on the nucleus to cytoplasm proxy framework described above (**Figure 2E**), we applied the dynamical model in scVelo to the melanoma cell compartment to reconstruct putative progression paths. Velocity vectors computed from nucleus- and cytoplasm-derived inputs indicated that a subset of cells exhibit directional trajectories pointing toward cluster 0 and cluster 3 (**Figure 3A**). However, latent time estimates revealed that cluster 3 corresponds to a more advanced (late) state while cluster 0 is positioned at an earlier stage of the trajectory; cluster 1 exhibits features consistent with a lower-differentiation or intermediate state (**Figure 3B**).

Spatial mapping of melanoma sub-clusters showed that cluster 3 cells are spatially aggregated within discrete tissue niches (**Figure 3C**). Correspondingly, velocity vectors suggest a directional bias in a subset of cells toward cluster 3, although heterogeneous orientations are observed across the population (**Figure 3D**). Differential expression analysis identified cluster-specific marker genes for the relevant groups; markers characteristic of cluster 1 and cluster 3 were subjected to pathway enrichment analysis using Metascape, which highlighted pathways including extracellular matrix degradation, cellular response to lipid, leukocyte activation, and Wnt signaling—processes that are plausibly linked to tumor invasion, metabolic adaptation, and immune modulation (**Figures 3E, F**).

Finally, we examined spliced/unspliced (cytoplasm/nucleus proxy) proportions for candidate prognostic genes and observed that *B2M* and *CD74* display elevated spliced-to-unspliced ratios and high expression within clusters 1 and 3 (**Figures 3G, H**), consistent with their putative involvement in later-stage or progressing malignant programs.

### 3.4 scVelo analysis uncovers dynamic programs of tumor-infiltrating T cells

**Figure 4:**
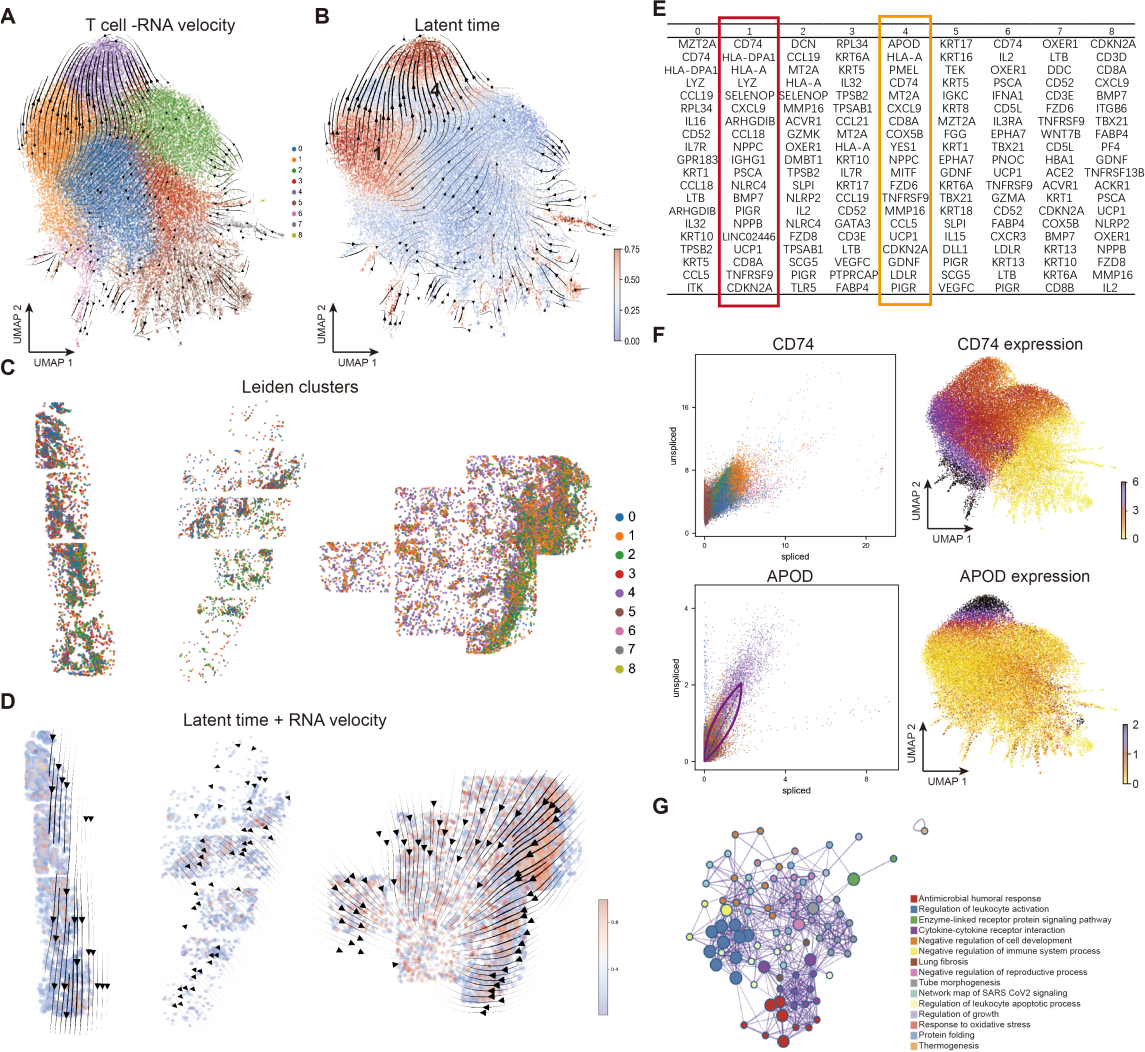
scVelo analysis showing distinct markers for T cell differentiation in melanoma tissues. **(A, B)** Leiden clustering **(A)** and latent time **(B)** in melanoma-infiltrating T cells. RNA velocity was calculated with the model established in **Figure 2E** and imposed on Leiden and latent time results. **(C)** Spatial distribution of melanoma-infiltrating T cells from distinct Leiden clusters. **(D)** Heatmap showing the spatial distribution of cells from distinct latent time. RNA velocity was calculated from scVelo and imposed on the latent time. **(E)** Tables showing markers for different Leiden clusters. **(F)** Dot plot showing spliced/unspliced levels in T cells and heatmap of marker gene expression for cluster 1 and cluster 4. **(G)** Metascape analysis showing the pathway enrichment with marker genes in Leiden cluster 1 and cluster 4.

We next applied the same spatial velocity pipeline to the T cell compartment. scVelo-derived velocity vectors indicated a predominant directional flow toward cluster 4, while a subset of cells also exhibited heterogeneous orientations across the embedding (**Figure 4A**). Latent time analysis further suggested that both cluster 1 and cluster 4 occupy relatively late positions along the inferred T cell trajectory, consistent with putative terminal or activated transcriptional states (**Figure 4B**).

Spatial projection of the T cell sub-clusters revealed relative enrichment of clusters 1 and 4 within specific tumor regions, although complete spatial segregation was not observed (**Figure 4C**). The spatial velocity field suggests a possible local transition structure among late-state T cell populations, but does not indicate a strictly linear progression from one cluster to another (**Figure 4D**). We further inspected transcript kinetics for T cell-expressed candidates and found that *CD74* and *APOD* show elevated expression in clusters 1 and 4. In addition, scVelo dynamical modeling suggests potential transcriptional activity changes reflected by the relative balance of spliced and unspliced signals, which may indicate future upregulation trends in these genes under the model assumptions (**Figures 4E, F**).

Further analysis with Metascape enrichment of cluster 1 and cluster 4 marker genes revealed immune-relevant processes, including regulation of leukocyte activation, cytokine–receptor interaction, and response to oxidative stress - pathways consistent with effector differentiation, signaling responsiveness, and metabolic/oxidative adaptation of tumor-associated lymphocytes (**Figure 4G**).

In addition, factors such as transcript stability, RNA export kinetics, and platform-specific detection efficiencies may influence the relative abundance of nuclear and cytoplasmic transcripts, potentially introducing bias into velocity estimation. We also observed gene-specific variability in subcellular transcript distribution (**Supplementary Figures 4A, B**), which may reflect biological regulation or technical factors and should be considered when interpreting gene-level dynamics. To evaluate the robustness of velocity inference to segmentation uncertainty, we introduced small perturbations to nuclear and cytoplasmic transcript counts and recomputed velocity. The inferred latent time showed strong concordance with the original results (Pearson correlation R = 0.954), indicating that the overall velocity structure is stable to moderate perturbations in compartment assignment (**Supplementary Figure 4C**). To evaluate robustness with respect to gene selection, we compared velocity outputs using different numbers of highly variable genes (100 and 500 genes). Latent time estimates showed strong consistency across settings, with a Pearson correlation of R = 0.921 (**Supplementary Figure 4D**). These results indicate that the inferred trajectories are not driven by a specific gene subset and remain stable under moderate variations in feature selection.

### 3.5 Dynamic marker genes associate with patient prognosis and form interaction networks

**Figure 5:**
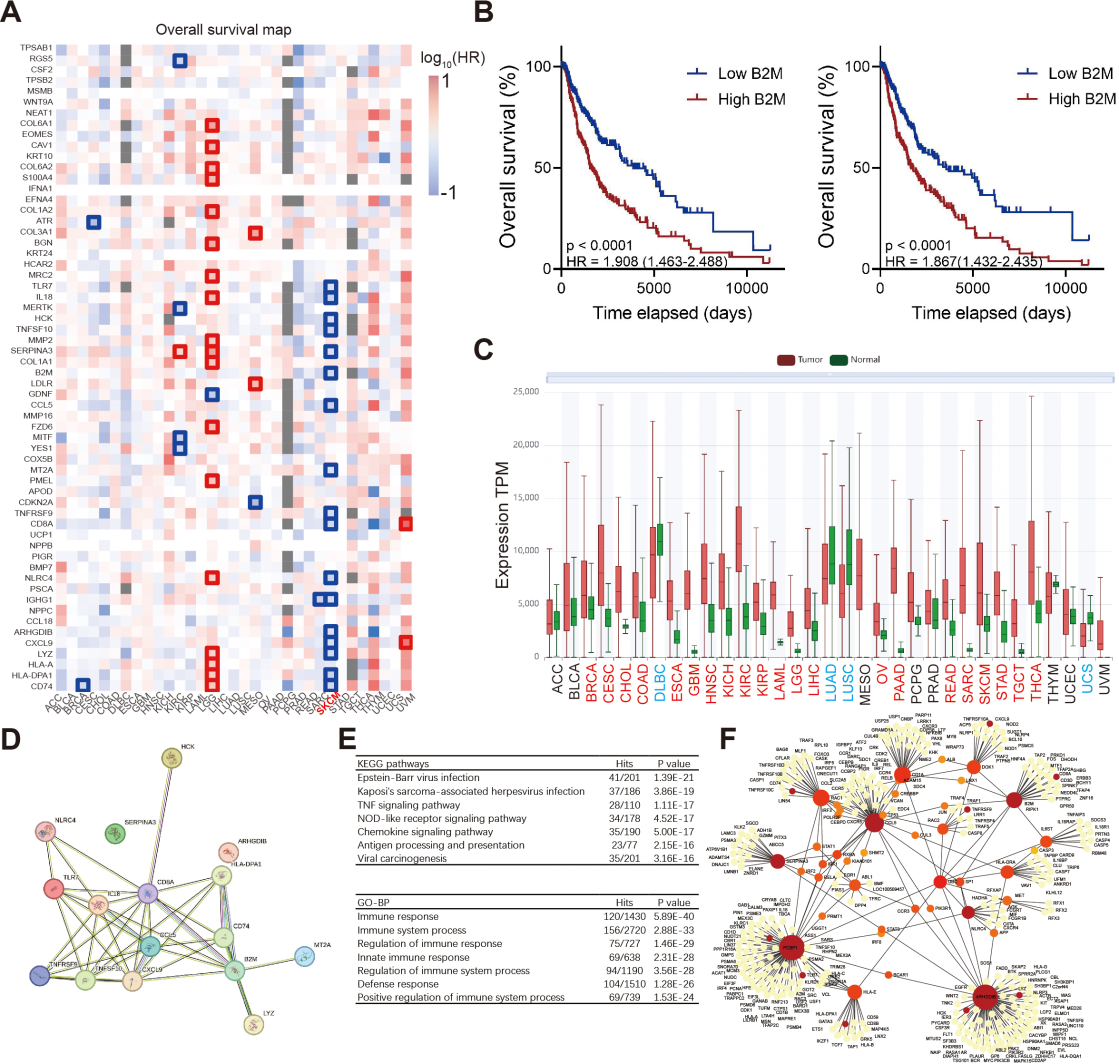
Prognostic marker genes for melanoma patients. **(A)** Survival map showing the hazard ratio in overall survival of marker genes identified from melanoma and infiltrating T cells across distinct cancer types in Gepia2 (http://gepia2.cancer-pku.cn/#survival). **(B)** Kaplan-Meier analysis showing the overall survival of melanoma patients with high vs. low B2M and CD74 levels. **(C)** Expression of CD74 across distinct cancer types and normal tissues. **(D)** Protein-protein interaction results using the prognostic melanoma markers in String (https://string-db.org/). **(E)** Tables showing the enriched KEGG pathway and GO-BP. **(F)** Network analysis results using melanoma prognostic markers in NetworkAnalyst (https://networkanalyst.ca/).

To evaluate the clinical relevance of the dynamic marker genes identified from melanoma cells and infiltrating T cells, we assembled the union of candidate genes and assessed their prognostic associations across TCGA cohorts (>20 cancer types). Pan-cancer survival screening showed that many of these genes exhibit prognostic associations in several malignancies, with the strongest and most consistent effects observed in cutaneous melanoma (**Figure 5A**).

We highlight two representative genes, *B2M* and *CD74*, for which Kaplan–Meier analysis stratified by high versus low expression revealed significant differences in overall survival among melanoma patients (**Figure 5B**). In addition, *CD74* is broadly overexpressed in multiple tumor types relative to normal tissues (**Figure 5C**), suggesting a wider role in tumor-immune biology.

To explore functional connectivity among the candidate markers, we constructed a protein–protein interaction (PPI) network using STRING and visualized the resulting interactome (**Figure 5D**). KEGG and GO–BP enrichment of the marker set identified pathways related to antigen processing and presentation, leukocyte activation, chemokine signaling, and innate immune responses (**Figure 5E**). Finally, network analysis (17) using NetworkAnalyst recapitulated central hubs and subnetworks that link immune modulators with antigen-presentation and signaling nodes (**Figure 5F**). The full list of marker genes used in these analyses is: ARHGDIB, B2M, CCL5, CD3E, CD74, CD86, CD8A, CXCL9, HCK, HLA-DPA1, HLA-E, IL18, LYZ, MR1, MT2A, NLRC4, SERPINA3, TLR7, TNFRSF9, TNFSF10, TYROBP.

Collectively, these results connect spatially inferred transcriptional dynamics to clinically relevant biomarkers and functional interaction networks, underscoring the potential of spatial RNA-velocity to uncover prognostic signals within tumor ecosystems.

### 3.6 CD74 depletion suppresses malignant behavior in melanoma cells

Our spatial RNA-velocity and prognostic analyses identified CD74 as a dynamic marker associated with advanced melanoma states and poor patient survival. CD74 was selected as a representative candidate to experimentally validate the predictive power of our spatial RNA velocity framework, rather than as the sole prioritized functional driver among identified prognostic markers.

**Figure 6:**
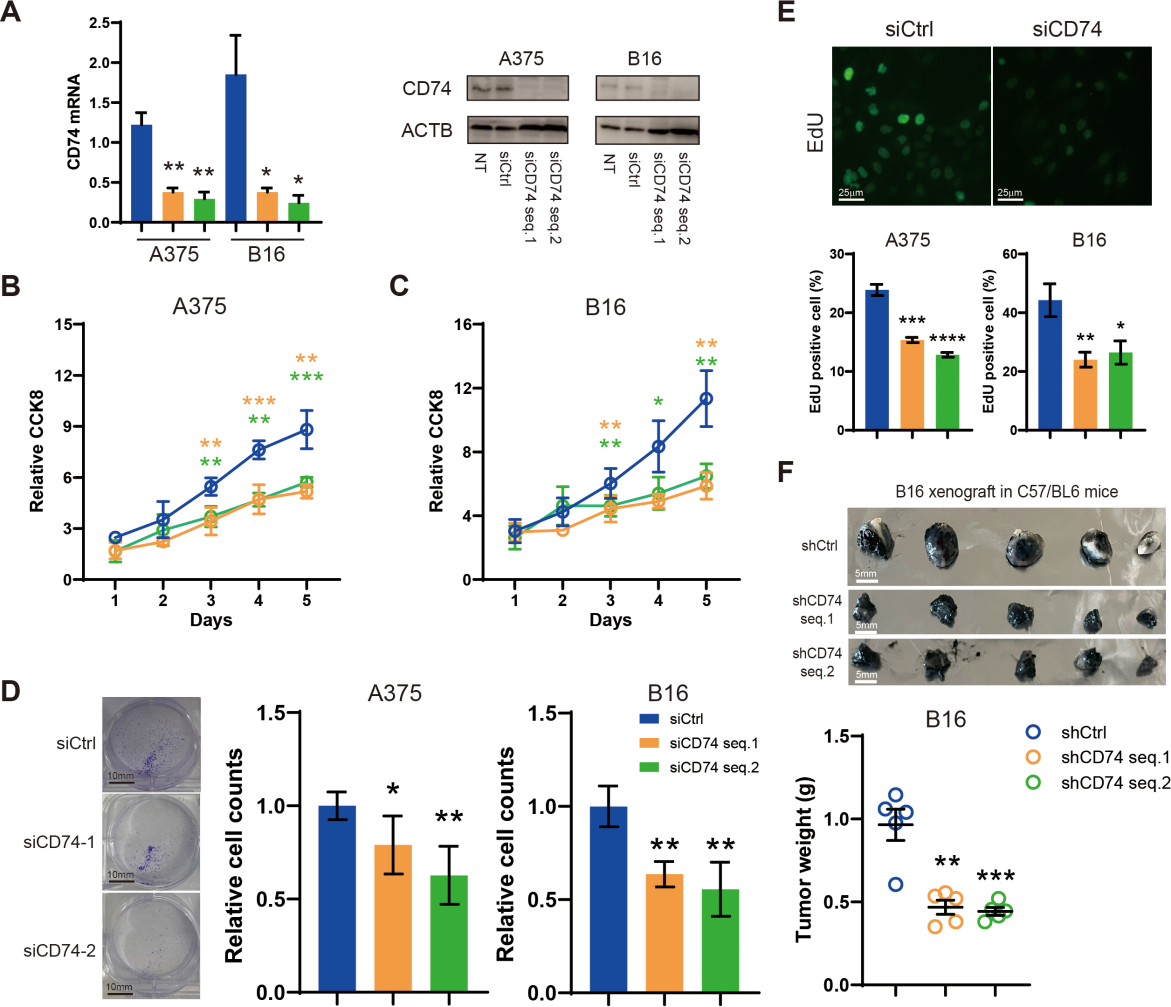
CD74 depletion suppressed malignant behavior in melanoma cells. **(A)** qPCR and immunoblot results showing the siCD74 efficacy in A375 and B16 cell lines (n = 3 in qPCR). **(B–C)** CCK8 results showing the proliferation of melanoma cells after CD74 depletion with siRNA or non-targeting control. n = 4 in each group. **(D)** Quantification of cell counts in melanoma cell lines after CD74 depletion with siRNA or non-targeting control in the transwell experiments. n = 3 in each group. **(E)** Typical figures and quantifications of EdU positive cells after CD74 depletion with siRNA or non-targeting control. n>50 cells per field, 3 replicates in each group. **(F)** *In vivo* experiments showing the proliferation of B16 cells in C53BL/6 mice after 16 days. Cells were transfected with CD74 shRNA or non-targeting control. n = 5 mice in each group. Data are presented as averages +/- SEM from independent experiments. *P<0.05, **P < 0.01, ***P < 0.001, using unpaired two-sided Students’ t-test.

To functionally validate its role in melanoma pathogenesis, we performed a series of *in vitro* and *in vivo* experiments. We first confirmed the efficacy of CD74-targeting siRNA (siCD74) in both human A375 and murine B16 melanoma cell lines, demonstrating significant knockdown at both the mRNA and protein levels (**Figure 6A**). Depletion of CD74 markedly suppressed the proliferative capacity of these cells, as evidenced by CCK-8 assays (**Figures 6B, C**) and direct cell counting (**Figure 6D**). Furthermore, transwell assays revealed that CD74 knockdown significantly impaired the invasive potential of melanoma cells (**Figure 6D**). To delve into the mechanism underlying the proliferation defect, we employed EdU incorporation assays and found that the percentage of cells actively replicating their DNA was substantially reduced upon CD74 silencing (**Figure 6E**), indicating an arrest in cell cycle progression. Finally, to substantiate these findings in a more physiologically relevant context, we injected B16 cells transfected with either siCD74 or a non-targeting control into C57BL/6 mice. After 16 days, tumors formed by CD74-depleted cells exhibited significantly less proliferation *in vivo* (**Figure 6F**, **Supplementary Figure 5**). Collectively, these functional studies demonstrate that CD74 is critical for sustaining the proliferation, invasion, and DNA replication of melanoma cells, thereby validating its pro-tumorigenic role predicted by our spatial dynamic model.

## 4 Discussion

In this study we present a spatial RNA velocity framework that extends transcriptional dynamics inference to spatial transcriptomic data by leveraging subcellular transcript localization (18). Applied to melanoma tissues, this approach reveals structured malignant progression programs and dynamic states of tumor-infiltrating T cells within their native spatial context.

In melanoma cells, spatial velocity analysis reveals a structured and continuous transcriptional landscape, characterized by coordinated progression across malignant states within the tumor microenvironment. The inferred latent time and velocity fields consistently support the presence of heterogeneous but organized tumor evolution rather than discrete, fully separated cell states.

In the immune compartment, T cells exhibit heterogeneous and dynamic transcriptional states, suggesting non-linear transitions associated with activation-related programs within the tumor microenvironment. Several dynamic marker genes identified in this study, including B2M and CD74, are associated with patient survival and form interaction networks enriched for immune-related pathways. Functional experiments further support a role for CD74 in melanoma biology. However, these associations are correlative and require further validation in independent cohorts and clinical settings. Notably, CD74 and B2M are interferon-responsive genes, which may reflect both tumor-intrinsic and microenvironmental signaling.

Methodologically, our nucleus to cytoplasm proxy strategy leverages subcellular localization information available from high-plex spatial imaging platforms (e.g., CosMx SMI) to approximate transcriptional kinetics in the absence of intronic/spliced read counts (19). This provides a complementary strategy to existing spatial trajectory inference methods. Nevertheless, results may be influenced by model assumptions and data quality, and should be interpreted as population-level trends rather than precise kinetic measurements.

Despite the strengths of our spatial RNA velocity framework, several limitations should be acknowledged. The nucleus-cytoplasm strategy is an approximation of the canonical unspliced-spliced RNA paradigm, and RNA localization can vary across genes and cell types. In particular, spliced transcripts may be retained in the nucleus, whereas incompletely processed transcripts may occasionally appear in the cytoplasm. Therefore, nuclear-cytoplasmic partitioning provides only an indirect proxy of RNA processing kinetics, and velocity estimates should be interpreted as coarse-grained directional trends rather than precise kinetic rates.

RNA velocity inference itself has inherent methodological limitations. Previous studies have shown that velocity estimates can be sensitive to noise, modeling assumptions, and data sparsity, and may become unstable under certain conditions (20–22). Nevertheless, we observed high concordance of latent time estimates across gene sets (100 vs. 500 genes, R = 0.921), supporting the robustness of global trajectory structure in our framework.

At the gene level, scVelo-derived outputs should be interpreted as probabilistic signals rather than deterministic kinetic measurements, particularly under proxy-based assumptions, and are most reliable when aggregated at the population level. In addition, segmentation-based assignment of subcellular transcripts introduces potential uncertainty in nuclear-cytoplasmic classification. Finally, the limited availability of high-resolution spatial datasets and the absence of matched intron-resolved scRNA-seq or spatial transcriptomic data currently preclude direct validation of the nucleus-cytoplasm proxy assumption, highlighting the need for future multi-modal validation.

In summary, we present a conceptually straightforward and broadly applicable framework to infer transcriptional dynamics from high-resolution spatial transcriptomics by exploiting subcellular transcript localization as kinetic proxies. Applied to melanoma, this spatial-dynamic view identifies spatially patterned malignant progression, T cell differentiation programs, and candidate prognostic markers embedded within interaction networks. The subsequent functional confirmation of CD74’s role in promoting melanoma malignancy not only validates our computational predictions but also underscores the potential of this integrated approach to uncover biologically and therapeutically relevant insights. While further technical validation is required, the approach provides a new lens to connect tissue architecture, cellular kinetics and clinical relevance, and it may facilitate the identification of spatially localized therapeutic vulnerabilities in melanoma and other solid tumors.

## Data Availability

The original contributions presented in the study are included in the article/**Supplementary Material**. Further inquiries can be directed to the corresponding author.
