# Supplementary material for: Spatial RNA velocity reveals cellular state transitions and prognostic markers in the melanoma microenvironment
Source: Front Oncol. 2026 Jun 22;16:1845013. doi: 10.3389/fonc.2026.1845013 (PMC13333528; doi:10.3389/fonc.2026.1845013)
Supplement: Supplementary file 1 [file DataSheet1.docx]

**Supplementary figures**

**Supplementary figure 1. Different cell types in melanoma tissues.**


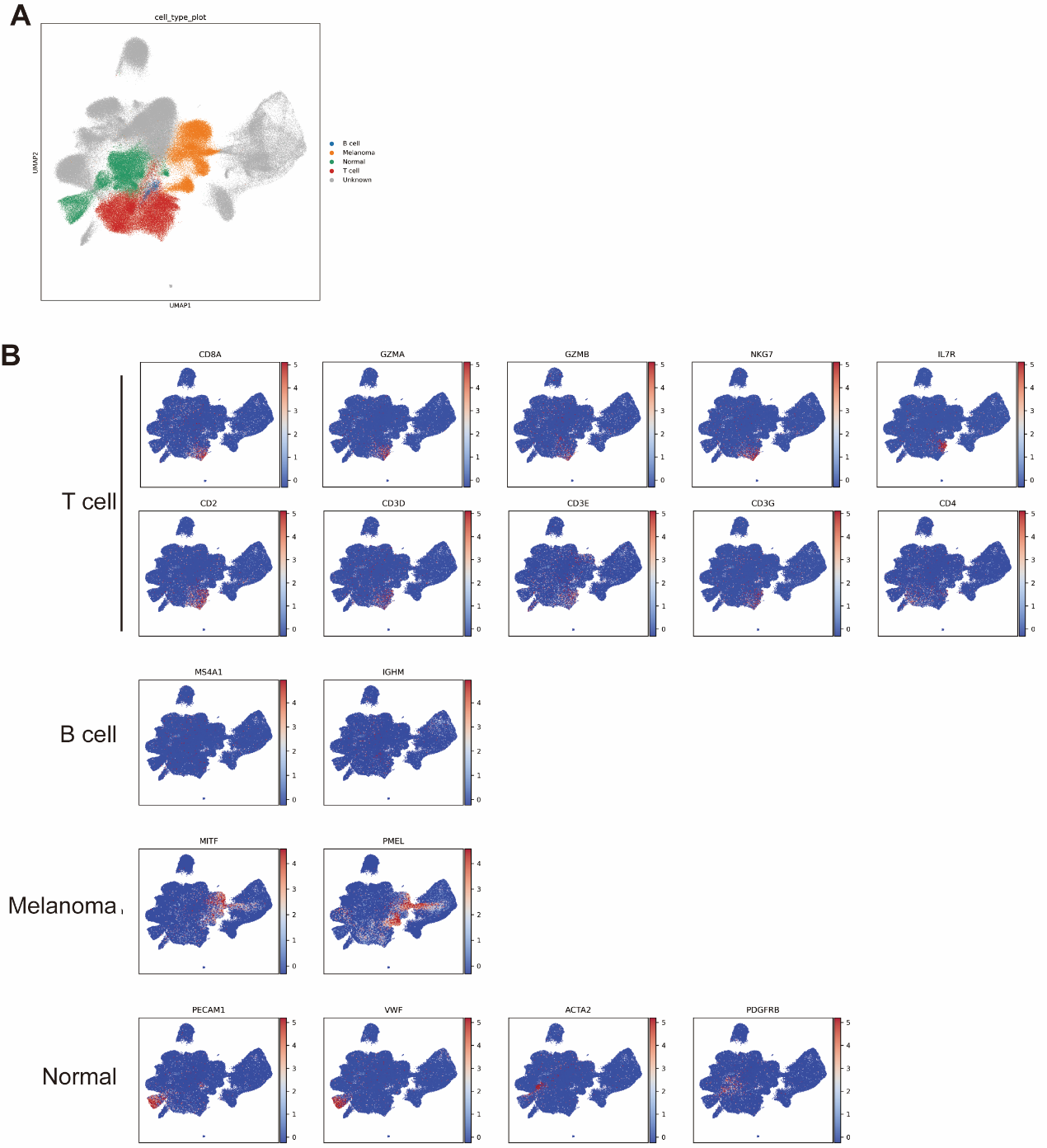
 (A) Leiden clustering results in melanoma tissues. T cells, B cells and melanoma cells were labelled. (B) Heatmaps showing the expression of distinct markers for T cells, B cels melanoma cells and normal skin cells in melanoma tissues.

**Supplementary figure 2. Spatial distribution of different cell types in melanoma tissues.**
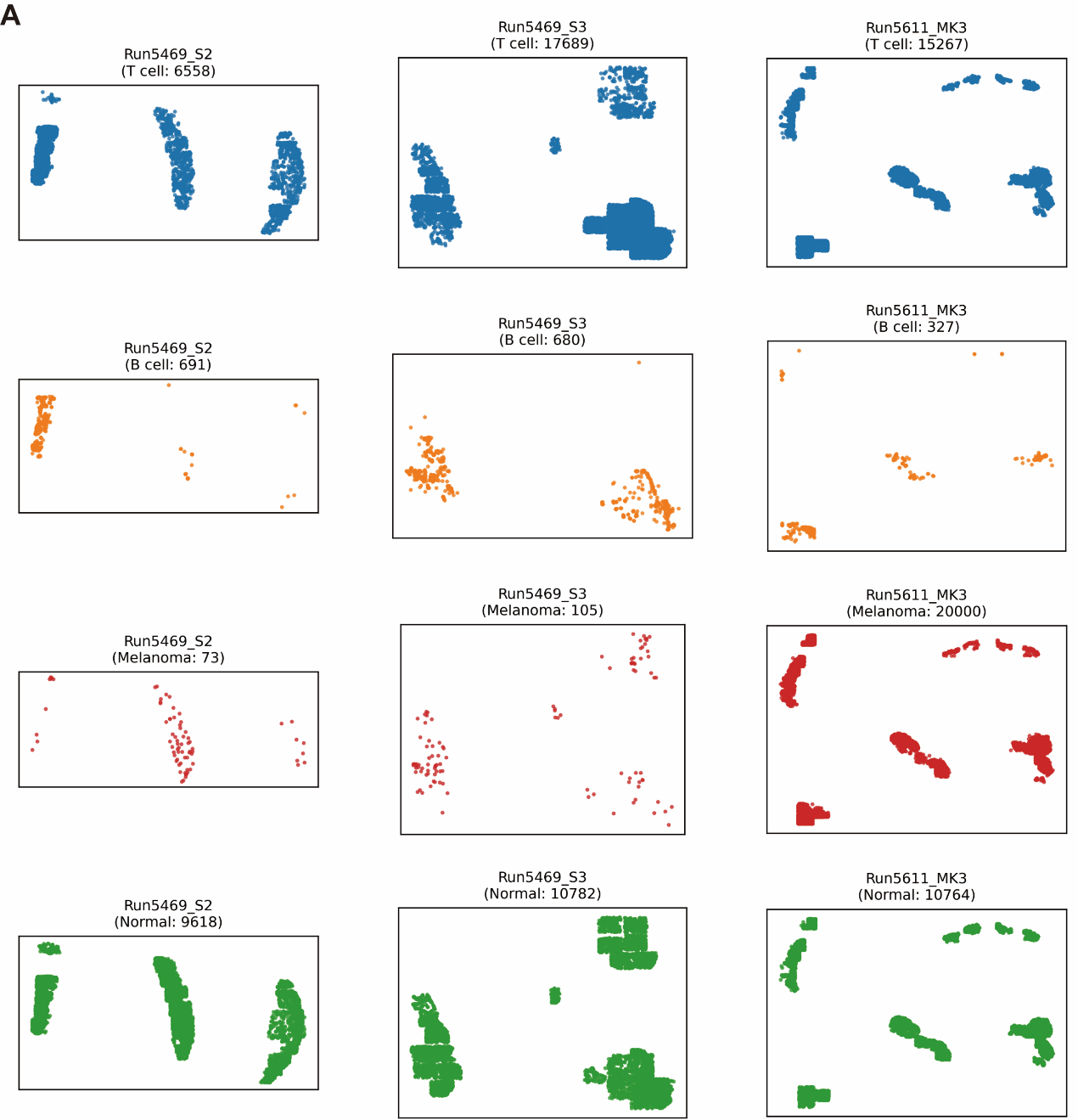


1. Spatial distribution of T cells, B cells and melanoma cells in melanoma tissues.

**Supplementary figure 3. Marker genes for melanoma cells and T cells.**


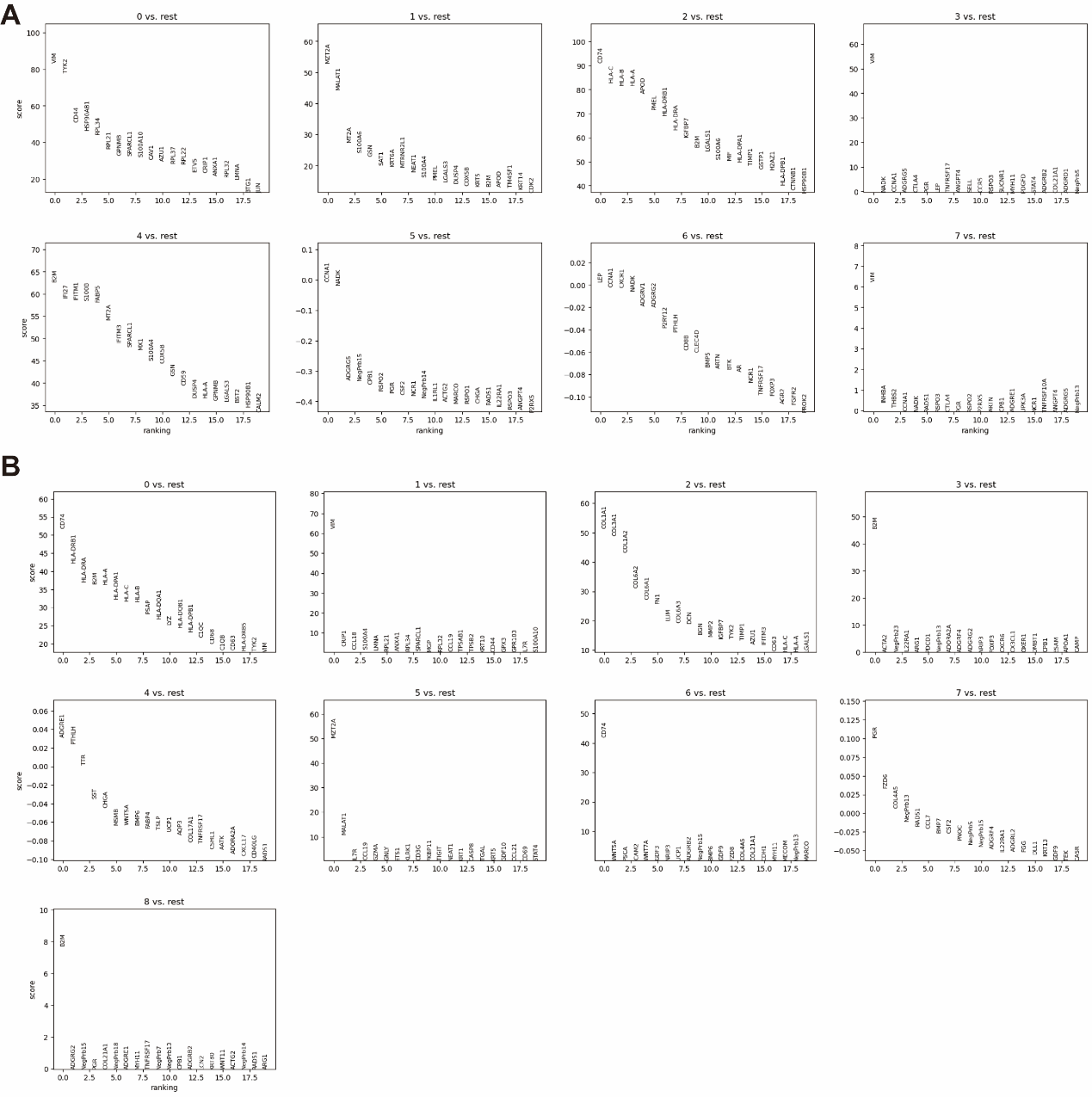
 (A-B) Scores for marker gene selection across different Leiden clusters in melanoma cells (A) and T cells (B).

**Supplementary figure 4. Distribution of cyto-nuclear ratio of transcripts.**


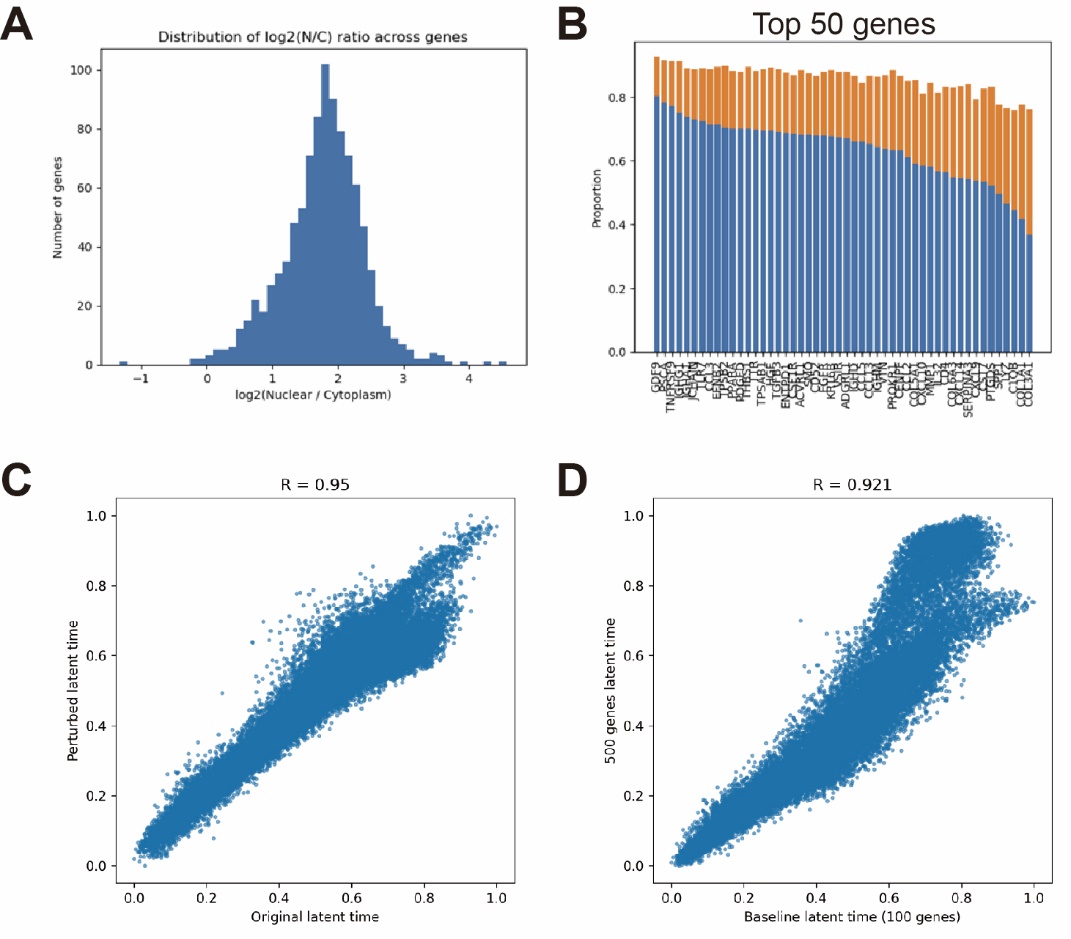


(A) Distribution of log2(nuclear/cytoplasm) of all transcripts in the melanoma tissues.

(B) Distribution of nuclear and cytoplasm proportion of top 50 most variated genes in the melanoma.

(C) Robustness of velocity inference under perturbations of compartment assignment. Random perturbations were introduced to nuclear and cytoplasmic transcript counts, and velocity was recomputed. Latent time estimates remained highly consistent with the original results.

(D) Robustness of velocity inference with respect to gene selection. Latent time estimates were compared between models using 100 and 500 highly variable genes, showing strong consistency across gene sets.

**Supplementary figure 5. CD74 depletion suppressed tumor growth and promoted survival of melanoma.**


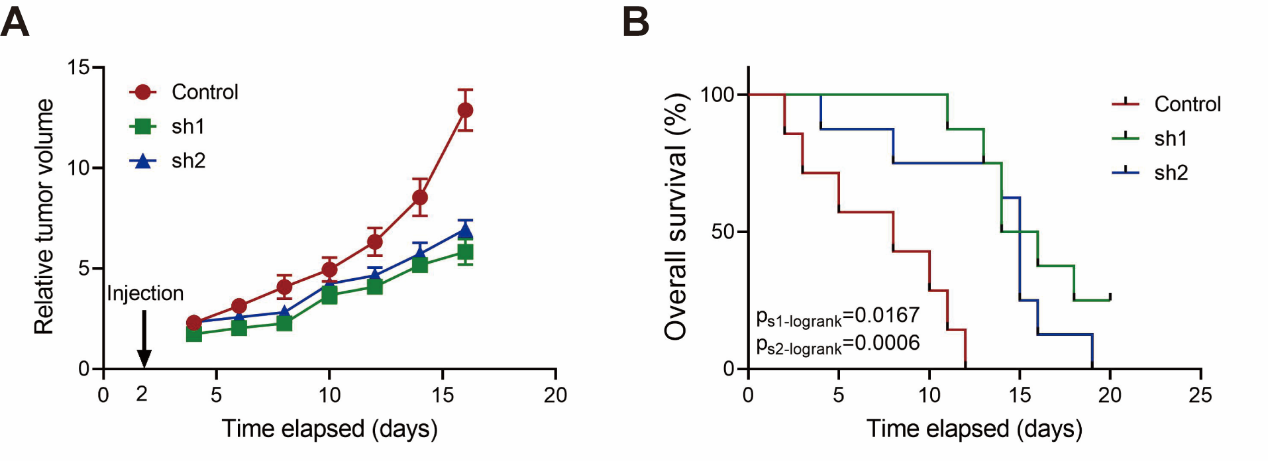


(A) Quantification of melanoma tumor size (tumor volume) with time. Cells stably expressing control or shCD74 were injected on day 2 and followed until day 16.

(B) Kaplan-Meier analysis of mice xenograft model. Mice were randomly split into 3 groups, 5 mice each and were followed 21 days since the injection of B16 cells.
